# 老年血液病患者单倍体造血干细胞移植后的死亡原因分析

**DOI:** 10.3760/cma.j.cn121090-20260125-00057

**Published:** 2026-07

**Authors:** 薇 赵, 瑞 马, 雪宜 罗, 葳 孙, 伟 韩, 晓冬 莫, 昱 王, 晓辉 张, 兰平 许, 晓军 黄, 于谦 孙

**Affiliations:** 1 北京大学人民医院血液科，北京大学血液病研究所，北京 100044 Peking University People's Hospital, Peking University Institute of Hematology, Beijing 100044, China; 2 国家血液系统疾病临床医学研究中心，血液肿瘤细胞与基因治疗北京市重点实验室，北京 100044 National Clinical Research Center for Hematologic Disease, Beijing Key Laboratory of Hematopoietic Stem Cell Transplantation, Beijing 100044, China

**Keywords:** 死因分析, 老年, 单倍体造血干细胞移植, The cause of death, Elderly, Haploidentical stem cell transplantation

## Abstract

**目的:**

分析老年血液病患者单倍体造血干细胞移植（haplo-HSCT）后的死亡原因。

**方法:**

本研究为单中心回顾性队列研究。选取2019年1月3日至2023年12月14日期间在北京大学人民医院接受haplo-HSCT的404例55岁以上患者，对其中死亡的患者进行死亡原因分析。

**结果:**

随访截至2025年10月11日，中位随访时间为708（12～2 343）d，其中共有139例在移植后死亡，全因死亡率为34.4％。在死亡患者中，死因为复发、感染、移植物抗宿主病（GVHD）的占比分别为40.3％、33.8％、11.5％。进一步亚组分析提示，髓系恶性肿瘤患者的主要死因为复发，而淋巴系统恶性肿瘤患者最常见的死因为感染；非缓解状态及可检测残留病阳性患者的主要死因为复发；患者年龄、造血干细胞移植共病指数、供者特异性抗体状态、预处理强度、干细胞来源及移植后不同阶段等因素未对死因分布产生显著影响。

**结论:**

复发及感染仍是老年血液病患者haplo-HSCT后的主要死亡原因。

## Abstract (en)

**Objective:**

To analyze the causes of death in elderly patients after haploidentical stem cell transplantation（haplo-HSCT）.

**Methods:**

This single-center retrospective cohort study included 404 patients aged >55 years who underwent haplo-HSCT at Peking University People's Hospital between January 3, 2019, and December 14, 2023, and analyzed the causes of death among those who died.

**Results:**

The last follow-up was October 11, 2025, with a median follow-up duration of 708（12–2 343）days. A total of 139 patients died after transplantation, with a mortality rate of 34.4％. Relapse was the leading cause of death（40.3％）, followed by infection（33.8％）and graft-versus-host-disease（11.5％）. Subgroup analysis revealed that relapse was the primary cause of death in patients with myeloid malignancies, whereas infection was most common in those with lymphoid malignancies. Patients in non-remission and those with minimal residual disease-positive status exhibited higher relapse death rates, representing the predominant cause of death. Factors such as patient age, HCT-CI, donor-specific antibody status, conditioning regimens, stem cell source, and post-transplantation phases did not significantly influence the causes of death.

**Conclusion:**

Relapse and infection remain the primary causes of death in older patients following haplo-HSCT.

异基因造血干细胞移植是老年血液病患者的重要治疗手段，可显著改善老年血液病患者的生存[1]–[4]。在缺乏同胞全合供者及非血缘供者时，单倍体供者成为重要的选择。近年来，随着移植技术的不断进步，接受单倍体造血干细胞移植（haplo-HSCT）的老年血液病患者的总生存（OS）获得显著改善，1年OS率可高达60％以上。尽管如此，仍有30％～40％的患者最终死亡。而移植后死亡的原因不尽相同，包括复发死亡和由感染、移植物抗宿主病（GVHD）、出血、器官衰竭等多种原因导致的移植相关死亡[5]。欧洲血液和骨髓移植协会（EBMT）数据显示，60岁以上急性髓系白血病（AML）/骨髓增生异常肿瘤（MDS）患者的4年移植相关死亡率（TRM）高达39％[6]；国际血液和骨髓移植研究中心（CIBMTR）的研究结果则显示，该人群2年TRM为32％～39％[7]。EBMT研究中60岁以上AML患者的1年TRM仍超过25％[8]；国内针对≥55岁急性白血病（AL）/MDS患者的生存分析显示1年TRM为23.3％[9]。分析死亡原因对改善老年患者的生存极为重要，本研究对北京大学血液病研究所≥55岁、haplo-HSCT后死亡的患者进行死因分布分析。

## 病例与方法

1. 病例：本研究为回顾性队列研究，在北京大学人民医院开展。纳入标准为：①于2019年1月3日至2023年12月14日期间在北京大学人民医院首次接受haplo-HSCT；②年龄≥55岁。本研究遵循《赫尔辛基宣言》，研究方案已获得北京大学人民医院伦理委员会批准（批件号：2026PHB481），豁免获取患者的知情同意。

2. 定义：复发死亡定义为移植后原发病复发或进展直接导致的死亡。TRM指除原发病复发外任何与移植相关因素导致的死亡发生率。主要死因判定参照国际通用的死因分类标准[10]–[11]。GVHD相关死亡是指在发生移植相关死亡的患者中患者的直接死因归于GVHD本身或其并发症，如器官衰竭，而非其他非GVHD相关的原因。感染相关死亡指发生移植相关死亡的患者中患者的直接死因归于活动性感染或其严重并发症，而非其他非感染性原因。器官衰竭相关死亡指死于心、肝、肾、肺等器官衰竭，除外感染或GVHD诱因。

急慢性GVHD根据国际标准进行诊断[12]–[13]。完全缓解（CR）定义为骨髓形态学原始细胞比例低于5％，无白血病相关发育异常，且无髓外白血病表现。OS期定义为从造血干细胞移植后首日起至因任何原因导致死亡的时间。无病生存（DFS）期定义为从移植之日起至疾病复发或死亡（以先发生者为准）的时间。复发定义为CR后外周血再现原始细胞或骨髓原始细胞>5％，或髓外任何部位出现白血病细胞浸润。

可检测残留病（MRD）采用流式细胞术（FCM）与实时定量聚合酶链反应（PCR）进行检测，FCM阳性指移植前末次骨髓穿刺标本中存在异常白血病相关免疫表型（LAIPs）的细胞比例超过0.01％。PCR阳性定义为：①具有特异性融合基因的患者，融合基因>0.01％；②缺乏特异性融合基因的患者，WT1水平>1.0％或连续2次>0.6％。

3. 移植方案：本研究主要采用两种主要预处理方案：①白消安/氟达拉滨/环磷酰胺+抗胸腺细胞球蛋白（Bu/Cy/Flu+ATG）；②Bu/Cy+ATG[9],[14]。

GVHD的预防在ATG（2.5 mg·kg ^−1^·d^−1^）的基础上，联合应用环孢素、吗替麦考酚酯及短程甲氨蝶呤。采用前列地尔注射液预防肝静脉闭塞症；复方新诺明预防耶氏肺孢子菌感染；伏立康唑或泊沙康唑预防真菌感染；阿昔洛韦预防单纯疱疹病毒及水痘-带状疱疹病毒感染，来特莫韦预防巨细胞病毒（CMV）感染。此外，每周两次通过实时PCR对CMV和EB病毒DNA载量进行监测。

4. 随访：本研究采用病例报告表（CRF）进行随访，末次随访时间为2025年10月11日。

5. 统计学处理：连续变量采用中位数及范围进行描述；分类变量采用频数及百分比（％）进行描述。连续变量的比较采用Mann-Whitney *U*检验，分类变量的比较采用*χ*^2^检验和Fisher精确检验。生存分析采用Cox回归模型，绘制Kaplan-Meier曲线，OS、DFS采用Log-rank检验比较。移植相关死亡及累积复发率采用竞争风险模型评估。复发和移植相关死亡互为竞争事件，死亡和无GVHD的复发为GVHD的竞争事件。死因构成比等可视化图表采用Adobe illustrator 2026绘制。统计分析采用SPSS 26.0及R 4.4.2（http://www.r-project.org）完成，设定双侧*P*<0.05为差异具有统计学意义。

## 结果

一、患者特征

共404例患者纳入该研究。中位随访时间为708（12～2 343）d。截至末次随访日，共139例患者在移植后死亡，患者的临床特征详见表1。移植后2年OS率为71.9％（95％*CI*: 67.4％～78.4％），2年DFS率为66.8％（95％*CI*: 62.2％～71.8％）。移植后100 d、180 d、1年及2年的TRM分别为6.5％（95％*CI*: 4.4％～9.2％）、13.1％（95％*CI*: 10.0％～16.6％）、19.4％（95％*CI*: 15.6％～23.4％）、21.3％（95％*CI*: 17.3％～25.5％）。2年累积复发率为12.8％（95％*CI*: 9.6％～16.4％），100 d急性GVHD发生率为35.5％（95％*CI*: 30.8％～40.1％），2年慢性GVHD发生率为41.8％（95％*CI*: 36.4％～47.2％）。

**表1:** 404例接受单倍体造血干细胞移植的老年血液病患者的基线特征

特征	总体（404例）	死亡患者（139例）
患者年龄［岁，*M*（范围）］	59（55～74）	59（55～74）
患者年龄分布［例（％）］		
55～59岁	225（55.7）	71（51.1）
60～64岁	139（34.4）	50（36.0）
65～69岁	31（7.7）	13（9.4）
≥70岁	9（2.2）	5（3.6）
患者性别［例（％）］		
女性	152（37.6）	54（38.8）
男性	252（62.4）	85（61.2）
供者-受者性别匹配［例（％）］		
女性供男性	169（41.8）	29（20.9）
其他	235（58.2）	110（79.1）
供者-受者亲缘关系［例（％）］		
子女供父母	364（90.0）	123（88.4）
旁系	40（10.0）	16（11.6）
供者-受者ABO血型关系［例（％）］		
相合	205（50.7）	74（53.2）
大不合	91（22.5）	27（19.4）
小不合	84（20.8）	29（20.9）
双向不合	24（5.9）	9（6.5）
诊断［例（％）］		
AML	189（46.8）	73（52.5）
MDS	122（30.2）	19（13.7）
ALL	66（16.3）	36（25.9）
其他^a^	25（6.7）	11（7.9）
诊断至移植的时间间隔［例（％）］		
<6个月	170（42.1）	52（37.4）
≥6个月	234（57.9）	87（62.6）
DSA［例（％）］		
阳性	64（15.8）	24（17.3）
阴性	339（83.9）	115（82.7）
MRD（仅AL/MDS）［例（％）］		
阳性	127（33.6）	50（39.1）
阴性	251（66.4）	78（60.9）
移植前疾病状态（仅AL）［例（％）］		
完全缓解	219（85.5）	68（73.9）
非完全缓解	37（14.5）	24（26.1）
HCT-CI［例（％）］		
0～2分	341（84.4）	117（84.2）
≥3分	63（15.6）	22（15.8）
预处理方案［例（％）］		
Bu/Cy/Flu+ATG	358（88.6）	122（87.8）
Bu/Cy+ATG	46（11.4）	17（12.2）
移植物来源［例（％）］		
PB	338（83.7）	112（80.6）
BM+PB	66（16.3）	27（19.4）
输注CD34^+^细胞［×10^6^/kg，*M*（范围）］	3.72（0.37～31.22）	3.72（0.37～31.22）
输注MNC［×10^8^/kg，*M*（范围）］	8.98（1.88～22.42）	8.99（5.10～18.16）

**注** AML：急性髓系白血病；MDS：骨髓增生异常肿瘤；ALL：急性淋巴细胞白血病；^a^包括慢性粒-单核细胞白血病、再生障碍性贫血、骨髓纤维化；AL：急性白血病；DSA：供者特异性抗体；MRD：可检测残留病；HCT-CI：造血干细胞移植共病指数；PB：外周血；BM+PB：骨髓联合外周血；MNC：单个核细胞；Bu：白消安；Cy：环磷酰胺；Flu：氟达拉滨；ATG：抗人胸腺细胞球蛋白

进一步分析不同年龄段患者的2年TRM，55～59岁组2年TRM为19.5％（95％*CI*: 14.4％～25.2％）；60～64岁组为22.4％（95％*CI*: 15.7％～29.8％）；65～69岁组为28.3％（95％*CI*: 12.9％～45.9％）；≥70岁组为17.4％（95％*CI*: 2.5％～59.5％）（*P*＝0.194）。

二、主要死因分析

在139例移植后死亡的老年患者中，56例（40.3％）死于疾病复发，83例（59.7％）为移植相关死亡。在移植相关死亡患者中，感染（33.8％）是最主要的死因，其次为GVHD（11.5％）和器官功能衰竭（4.3％）。其他死亡原因包括出血、移植失败、脑梗死及消化道出血等。

三、感染死因分析

1. 死亡时间：在139例死亡患者中，共47例死于感染。其中，有20例（14.4％）死亡发生在移植后100 d内，有32例（23.0％）发生在移植后1年内。

2. 感染部位：28例（20.1％）感染死亡患者死于呼吸道感染，12例（8.6％）患者死于脓毒症，5例（3.6％）患者死于中枢神经系统感染（1例为CMV脑炎）。

3. 病原菌：在47例死于感染的患者中，死于单纯细菌感染20例（42.6％），死于单纯真菌感染3例（6.4％），死于病毒感染2例（4.3％），均为EB病毒感染，死于混合感染13例（27.7％），感染原因不明者11例。

四、亚组分析

1. 患者年龄：对不同年龄段（55～59岁、60～64岁及≥65岁）患者的死亡原因进行比较分析，未发现差异有统计学意义（*P*＝0.948）。复发均为各组最主要的死亡原因，占比分别为40.8％、40.0％和38.9％；其次为感染，占比分别为33.8％、34.0％、33.3％；再次为GVHD，占比分别为12.7％、12.0％、5.6％（图1A）。

**图1:**
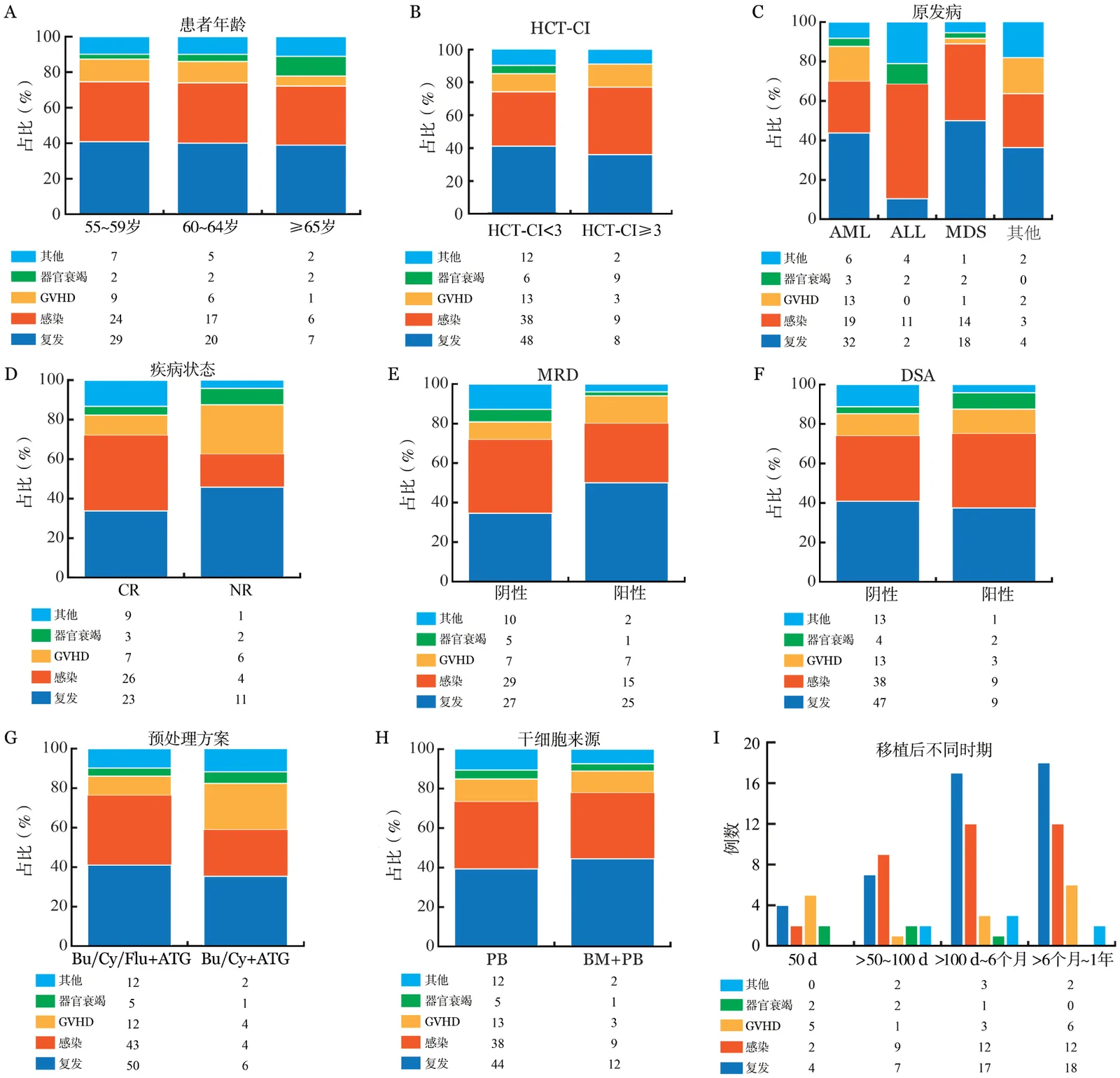
404例接受单倍体造血干细胞移植的老年血液病患者亚组死因分析 **A** 患者年龄；**B** 造血干细胞移植共病指数（HCT-CI）；**C** 原发疾病；**D** 移植前疾病状态；**E** 可检测残留病（MRD）；**F** 供者特异性抗体（DSA）；**G** 预处理方案；**H** 干细胞来源；**I** 移植后不同时期 **注** GVHD：移植物抗宿主病；AML：急性髓系白血病；ALL：急性淋巴细胞白血病；MDS：骨髓增生异常肿瘤；CR：完全缓解；NR：未缓解；Bu：白消安；Cy：环磷酰胺；Flu：氟达拉滨；ATG：抗人胸腺细胞球蛋白；PB：外周血；BM：骨髓

2. 造血干细胞移植共病指数（HCT-CI）：在139例患者中，117例（84.2％）HCT-CI <3分，22例HCT-CI ≥3分。两组复发及感染相关死亡占比较高，分别为41.0％对36.4％、32.5％对40.9％，GVHD相关死亡次之（11.1％对13.6％，*P*＝0.881）（图1B）。

3. 原发病：不同疾病患者的死亡原因存在差异。在髓系恶性肿瘤（包括AML和MDS）患者中，死亡首要原因为复发，其次为感染和GVHD；而在急性淋巴细胞白血病（ALL）患者中，感染为主要死因（57.9％），复发次之（10.5％），组间差异具有统计学意义（*P*＝0.006）（图1C）。

4. 移植前疾病状态：在92例AL患者中，68例移植前达到完全缓解（CR），24例为未缓解（NR）。CR组中复发死亡和感染死亡分别占33.8％和38.2％，其次为GVHD（10.3％）；NR组中以复发为主要死因，GVHD（25.0％）和感染（16.7％）次之（*P*＝0.841）（图1D）。

5. MRD：在128例AL/MDS患者中，移植前50例为MRD阳性，78例为MRD阴性。MRD阳性组的复发死亡率高于阴性组（50.0％对34.6％），感染相关病死率均较高（30.0％对37.2％），但差异未达统计学意义（*P*＝0.159）（图1E）。

6. 供者特异性抗体（DSA）：移植前115例患者为DSA阴性，24例为DSA阳性。复发死亡（40.9％对37.5％）和感染死亡（33.0％对37.5％）均占多数，组间差异无统计学意义（*P*＝0.652）（图1F）。

7. 预处理强度：绝大多数患者（122例，87.8％）接受Bu/Cy/Flu+ATG预处理方案，仅17例接受Bu/Cy+ATG方案。Bu/Cy/Flu+ATG组中，41.0％患者的死因归于复发，35.2％归于感染，9.8％归于GVHD；Bu/Cy+ATG组中35.3％死于复发，感染和GVHD各占23.5％（*P*＝0.391）（图1G）。

8. 干细胞来源：移植物来源为单纯外周血（PB）组与骨髓联合外周血（BM+PB）组在死亡原因方面差异无统计学意义（*P*＝0.992）。两组均以复发为首要死因（39.3％对44.4％），其次为感染和GVHD（感染：33.9％对33.3％；GVHD：11.6％对11.1％）（图1H）。

9. 移植后不同时期：在移植后50 d内，共计13例（9.4％）患者死亡，死于复发、感染、GVHD的分别有4例（2.9％）、2例（1.4％）、5例（3.6％）。在移植后50～100 d、100 d～6个月及6个月至1年内，感染及复发均为主要死因，其次为GVHD（图1I）。

## 讨论

本研究针对≥55岁老年haplo-HSCT人群系统分析了该群体死亡原因的分布特征。研究显示，疾病复发是主要死亡原因。在移植相关死亡中，死于感染占比最高，其次是GVHD和器官功能衰竭。

在EBMT的全年龄人群回顾性研究中，复发也是allo-HSCT后的主要死因[15]。此外，Zielińska等[16]和Metha等[17]的研究分别在中位年龄52和51岁的人群中开展，发现haplo-HSCT后主要死因为原发病复发或进展，与本研究结论相似。与年轻患者相比，老年患者的复发死亡占比更高[15]–[17]。老年白血病患者移植后复发率更高的原因可能包括：一方面，随着年龄的增加，异常克隆性造血增加；而且，年长供者中等分化潜能的克隆性造血（CHIP）增加，使得供者来源白血病的发病率增加，导致复发风险增加[18]–[19]。另一方面，预处理强度也会影响复发率[20]–[21]。对于老年患者，优先推荐采用减低毒性预处理方案，相比于常规清髓预处理，其器官毒性明显降低，尤其是对肾脏的毒性[22]，明显降低了TRM，但是以高复发率为牺牲[9]，[23]–[25]。有趣的是，ALL患者中复发为次要死因，感染为首位死因。原因可能为TKI时代和针对淋巴细胞的免疫治疗如贝林妥欧双抗、奥加因妥珠单抗以及嵌合抗原T细胞疗法显著改善了ALL患者的预后。在老年移植患者中合理应用包括移植后维持治疗、基于MRD指导的抢先干预策略等针对移植后复发防治的策略，是改善移植后无复发生存的重要措施。

感染是异基因造血干细胞移植患者的第二大死因，也是首要的移植相关死亡原因[17],[27]。既往研究也有相同结论[8],[16]–[17]。老年患者感染风险增高是多种因素共同作用的结果。高龄患者往往合并多种基础疾病及营养不良，可能伴随更为持久或严重的免疫缺陷；此外，有研究提示表观遗传学衰老等可能导致免疫重建迟缓，使得感染风险提高，最终导致感染相关死亡率升高[17],[27]。因此，加强老年haplo-HSCT患者的感染预防可能是未来提高老年患者移植疗效的措施之一。

GVHD是移植后的重要死亡原因。相比年轻人，老年患者发生GVHD风险更高[8],[17]。老年患者免疫重建迟缓、衰老的炎性宿主环境等均可促进GVHD的发生。JAK抑制剂（如芦可替尼）预防高危患者发生GVHD、间充质干细胞治疗难治性GVHD，以及输注调节性T细胞诱导免疫耐受等新策略，将为GVHD的防治提供更多可能。此外，需更好地平衡移植物抗白血病效应（GVL）与GVHD之间的关系，在保证GVL作用的同时，不增加GVHD发生风险[28]。

当然，本研究存在一些缺陷：首先，本研究为单中心回顾性研究，样本量相对有限；且随访时间较短，未能涵盖所有患者的最终死亡原因。其次，本研究将死亡原因定义为直接导致死亡的根本疾病。而终末期患者的死亡常存在多种因素共同作用，因此部分病例的死因分类有一定模糊性。未来需要开展多中心、前瞻性、长期随访的研究以进一步验证。

总之，haplo-HSCT技术的发展为许多老年恶性血液病患者带来了希望，但移植后复发、感染和GVHD仍是影响疗效与生存的关键因素。本研究对死亡原因分布的分析，有助于未来进一步改进老年移植策略，改善老年患者的生存。
